# Nurses’ Attitudes toward, and Needs for Online Learning: Differences between Rural and Urban Hospitals in Shanghai, East China

**DOI:** 10.3390/ijerph15071495

**Published:** 2018-07-15

**Authors:** Weijie Xing, Linjun Ao, Huiting Xiao, Li Cheng, Yan Liang, Junqiao Wang

**Affiliations:** 1 School of Nursing, Fudan University, Shanghai 200032, China; 14301170046@fudan.edu.cn (L.A.); 14301170030@fudan.edu.cn (H.X.); liangyan@fudan.edu.cn (Y.L.); junqwang@fudan.edu.cn (J.W.); 2 The Nethersole School of Nursing, The Chinese University of Hong Kong, Shatin, N.T., Hong Kong Special Administrative Region, Shatin, Hongkong 999077, China; lilycheng@cuhk.edu.hk

**Keywords:** nurse, online learning, continuing education, attitudes, needs, rural

## Abstract

Health professionals need continuing education to maintain their qualifications and competency. Online learning increases the accessibility and flexibility of continuing education. Assessment of nurses’ attitudes toward, and needs for, online learning can provide suggestions regarding learning program design and delivery. This study aimed to evaluate Chinese nurses’ attitudes toward, and needs for, online learning, and to explore the differences in attitudes and needs between nurses working in rural and urban hospitals. This work is a secondary analysis of a multicenter cross-sectional study conducted in Shanghai in 2015 (*n* = 550). Multiple regression techniques were used to determine the factors associated with nurses’ attitudes toward, and needs for, online learning. Results showed that nurses in rural hospitals had more positive attitudes toward online learning (102.7 ± 14.2) than those in urban hospitals (98.3 ± 12.9) (*p* < 0.001). For rural hospitals, nurses who could use computers and access the internet in their workplace reported more positive attitudes than those who could not. For urban hospitals, nurse educators showed significantly more positive attitudes than others. Communication skills (86.5%) and patient education (86.3%) were the most commonly-reported learning needs for nurses regardless of their working settings. Chinese nurses were willing to adopt online learning as a continuing education method. Nurses working in rural hospitals displayed more positive attitudes toward, and needs for, online learning than those working in urban hospitals. Nursing educators and managers should develop online learning programs and provide appropriate support to fulfill nurses’ learning needs, especially for those working in rural healthcare settings.

## 1. Introduction

Nurses, as the largest component of the health workforce, play critical roles in patient safety, patient education, treatment follow-up, disease prevention, and health promotion [1,2,3]. Nursing activities have increased healthcare accessibility, reduced patients’ symptoms, enhanced positive health outcomes, improved cost-effectiveness, and increased patient satisfaction regarding healthcare services [4,5,6]. In order to enhance skills, modify thinking and action, and maintain clinical competency, nurses need to continue learning throughout their careers to adapt to rapid changes in working environments [7,8]. 

Continuing nursing education has been recognized as a mandatory qualification requirement in most countries [9,10]. However, traditional continuing learning methods that usually use face-to-face classes as the major approach can be difficult for nurses to attend. Heavy workloads, time or energy constraints, geographic distance, learning costs, and lack of learning opportunities or support from supervisors were the key barriers to nurses’ participation in continuing education programs [11,12], especially for those working in rural and remote healthcare settings [13,14,15].

Evidence from many countries, including China, showed that individuals living in rural areas tend to have lower health confidence, a shorter life expectancy, more incidence of diseases, and a higher mortality rate than urban residents [16,17,18,19,20]. These rural–urban health outcome disparities are likely related to a range of factors, including education level, employment opportunities, poverty, and access to healthcare services. Nurses working in rural areas play a vital role in closing the health gap for the rural population [21]. Increasing their access to continuing education is necessary to maintain their competency to provide qualified health services for rural residents.

Online learning, also called e-learning, involves the combined use of technology and social networks [22]. Online learning is more flexible, accessible, convenient, and cost-effective; moreover, it increases learning opportunities and offers a more distinctive learning environment for nurses than traditional learning methods [23]. Online learning has been applied in nursing education in many studies, and demonstrated equal learning outcomes and greater satisfaction than traditional learning [23,24]. Learners’ characteristics, attitudes, and needs were critical factors affecting learning efficacy, outcomes, and satisfaction [25]. Therefore, online learning can be considered as an alternative method to bridge the gap between nurses’ learning needs and education services. The assessment of nurses’ attitudes toward, and needs for, online learning can provide suggestions for learning program design and delivery. 

The number of registered nurses in China reached 3.8 million at the end of 2017. However, nearly 40% of those held only a primary diploma [26]. Chinese nurses are confronted with the challenge of updating their knowledge to improve their competency. Although there are some studies reporting nurses’ perceptions of online learning in several countries [8,27,28,29], evidence from China is currently lacking. In addition, few comparative studies have examined the differences in attitudes or needs between nurses working in different health settings. 

Therefore, the main aim of this study was to reveal Chinese nurses’ attitudes toward, and needs for, online learning, and then to explore the differences in attitudes and needs between nurses working in rural and urban hospitals. The findings of this study will aid nurse managers and educators in different healthcare settings to provide appropriate support and resources to meet nurses’ continuing learning needs. 

## 2. Materials and Methods

### 2.1. Data Resources and Samples

This study was a secondary analysis of a multicenter cross-sectional study, which aimed to investigate nurses’ perceptions of e-learning. Original data were obtained by a self-reported, online questionnaire survey conducted in 2015 in Shanghai, China.

Participants were recruited through a stratified multistage sampling framework. First, eight hospitals were conveniently selected from eight main regions in Shanghai. Second, nurses were randomly recruited from each hospital by their staff identification number. The inclusion criteria of participants included nurses who have a formal registered license and have been working for more than half a year. Ethical approval was obtained from the institutional review board at the School of Nursing, Fudan University. Eligible nurses were invited to participate in the study, and informed consent was obtained prior to questionnaire administration. Questionnaires were inputted into an online survey platform, and quality criteria were established to identify missing items, logical contradiction, and completion time of less than 300 s. Participants received the questionnaire link and completed it via mobile phone or computer. All data were stored in the online survey platform, and visiting access was limited to the principal investigator. 

### 2.2. Measures

Nurses’ attitudes toward online learning were evaluated by a 28-item scale. This scale was developed by Malaysian researcher Chong [28]. The scale consisted of six subscales related to computer use (four items), convenience and flexibility (five items), interaction with facilitators and other students (seven items), access to knowledge (two items), positive learning experience (seven items), and improvement of nursing care (three items). Each item was scored by a five-point Likert scale from 1 (strongly disagree) to 5 (strongly agree). A sum of all items provided a total score ranging from 28 to 140. Scores over 100 indicated a positive attitude toward online learning. After obtaining permission from the original author, the questionnaire was translated from English to Simplified Chinese guided by World Health Organization guidelines [30]. The overall content validity index of the Chinese questionnaire was 0.96, and the Cronbach’s alpha was 0.93.

Nurses’ needs for online learning were measured by a multiple-choice question with nine options, which reflected learning needs for: Specialty nursing; general nursing; nursing skills; communication skills; patient education; nursing informatics; nursing education methods; nursing management methods; nursing research; and, evidence-based nursing. Participants could choose more than one answer from these options. 

Demographic and background characteristics were collected by a self-developed questionnaire with 10 items, including: Gender; age; marital status; educational level; hospital location; years of working experience as a registered nurse; job position; average monthly income; computer use; and, internet access at home and in the workplace. Shanghai is divided into 18 county-level divisions consisting of 17 districts and one county, of which 9 districts (i.e., Huangpu, Luwan, Xuhui, Changning, Jing’an, Putuo, Zhabei, Hongkou, and Yangpu) are located in the core of the city and referred to as urban areas. The remaining eight districts and one county (i.e., Baoshan, Minhang, Jiading, Jinshan, Songjiang, Qingpu, Fengxian, and Pudong districts, and Chongming County) are further away from urban areas. In this study, nurses working in hospitals in Xuhui, Jing’an, and Yangpu were defined as urban nurses, while nurses working in Pudong, Qingpu, and Minhang were defined as rural nurses.

### 2.3. Statistical Method

Data were managed and analyzed with SPSS^®^ for Windows version 24.0. (IBM, Armonk, NY, USA) Categorical variables were summarized as frequencies and percentages, and continuous variables as means and standard deviations. Bivariate and multivariate analyses were used to identify factors associated with attitudes toward online learning of nurses in rural and urban hospitals. In bivariate analyses, relationships of potential associated factors with nurses’ attitudes toward online learning were assessed by *t*-tests or chi-squared tests. Three regression models were built to explore the associated factors of nurses’ attitudes toward online learning with stratification of hospital area. All statistical tests involved were two-sided with level of significance (*p* value) set at 0.05.

## 3. Results

### 3.1. Demographic and Background Characteristics of Rural and Urban Nurses

A total of 534 nurses provided informed consent to participate and completed the questionnaire, with a response rate of 97.1%. The key demographic and background characteristics of participants by hospital location are presented in Table 1. 

Most participants were women (99.1%, *n* = 529), and their mean age was 29.9 (*SD* = 6.3) years. A total of 44.2% (*n* = 236) of participants worked in rural hospitals, while the remaining 55.8% (*n* = 298) worked in urban hospitals. The mean duration of years of working experience as a registered nurse was 8.1 (*SD* = 6.5) years. More nurses in urban hospitals had a Bachelor of Science degree or above (14.1%, *n* = 42) than those working in rural hospitals (7.2%, *n* = 17). In urban settings, a majority of participants (49.3%, *n* = 147) had an average monthly income of RMB 5000–8000 (approximately USD $750–1200), while in rural settings, a majority of nurses received an average monthly income of less than RMB 5000 (41.9%, *n* = 99). Regardless of the geographic location, almost all participants could use a computer to access the internet at home (98.3%, *n* = 525), but only 39.3% and 28.0% of urban and rural nurses, respectively, could use a computer to access the Internet in their workplace.

### 3.2. Attitudes Toward Online Learning of Rural and Urban Nurses

Table 2 shows the comparison of the total and subscale attitudes scores between rural and urban nurses. Overall, the average score for attitude was 100.2 (*SD* = 13.6), with a range from 38 to 135. The total attitude score of rural nurses (102.7 ± 14.2) was significantly higher than that of urban nurses (98.3 ± 12.9) (*p* < 0.001). Regarding subscale, rural nurses gave higher scores than urban nurses in four aspects, including convenience and flexibility, interaction with facilitators and students, positive learning experience, and improvement of nursing care. 

### 3.3. Association between Nurses’ Characteristics and Attitudes toward Online Learning 

The results of the univariate analysis of association between nurses’ characteristics and attitudes toward online learning are shown in Table 3. In rural hospitals, nurses’ positive attitudes were significantly associated with computer use and internet access at home and in their workplace. In urban hospitals, nurses over 30 years old, married nurses, nurses working over 10 years, and nurse educators, reported more positive attitudes toward online learning than the others. 

### 3.4. Regression Analysis of the Associated Factors of Nurses’ Attitudes toward Online Learning

Multiple linear regression analysis was performed to explore the associated factors identified by univariate analysis (Table 4). Gender, computer use, and internet access at home were not included in the model because nearly all participants were female and had computer and internet access at home. According to the results of the regression, location of hospital, computer use, and internet access in the workplace were significantly associated with nurses’ attitudes toward online learning. Furthermore, associated factors were different between rural and urban nurses. For rural hospitals, nurses who could use a computer and had internet access in their workplace reported significantly more positive attitudes than those who could not. For urban hospitals, nurse educators showed significantly more positive attitudes than the others.

### 3.5. Online Learning Needs of Rural and Urban Nurses

Table 5 displays the online learning needs of nurses. Regardless of geographic location, nurses reported the highest learning need for communication skills (89.4%), and the lowest need for nursing research and evidence-based nursing (70.6%). Although rural nurses reported more learning needs in almost all topics (i.e., specialty nursing, general nursing, nursing skills, communication skills, patient education, nursing management methods, nursing research, and evidence-based nursing), there were no statistically significant differences compared to urban nurses.

## 4. Discussion

To our knowledge, this multicenter survey was the first study to examine Chinese nurses’ attitudes towards, and needs for, online learning, and to compare the differences between rural and urban healthcare settings. This study found that rural nurses had more positive attitudes towards, and needs for, online learning than urban nurses. Nurses who have access to a computer and the internet in their workplace reported more positive attitudes than those who do not have access. Communication skills and patient education were the highest-ranked topics of interest for nurses regardless of the working location. These findings can aid nursing managers and educators in the development and delivery of successful and effective online learning programs designed to fulfill nurses’ needs in different healthcare settings. 

The average score for attitude was 100.2, which means nurses had a relatively positive attitude toward online learning. This was consistent with the results of previous studies in Taiwan [31], Malaysia [28], Turkey [8], and United Kingdom [32]. A global nursing shortage, high workloads, and lack of time, hinder nurses in their participation in continuing learning [10]. Online learning is flexible and convenient with respect to time and location, which can be helpful for nurses to balance their jobs, learning, and families. In addition, with the development of information technology and social media, massive open online courses have become popular in nursing education [33]. Increasing numbers of nurses have become familiar with the new learning style and obtained positive experiences from online learning.

The results of the regression showed that rural nurses displayed significantly more positive attitudes than those working in urban hospitals. Nurses working in rural healthcare settings usually have a lower initial education level than their urban counterparts. Thus, their need for continuing learning is much stronger than urban nurses [15]. However, their access to continuing education is usually inhibited by geographic distance, high cost, limited opportunities, low staffing levels, and work commitments [15]. One considerable advantage of online learning is the enhanced access for learners. After removing the constraint of commuting to a specific location at a certain time, nurses working in rural areas can have more time, energy, and opportunities, to attend continuing education. Thus, online learning may be helpful to maintain a skilled nursing workforce in rural health care facilities. 

We also found that nurses working in rural hospitals had limited access to computers and internet connections in their workplace, which negatively affected their attitudes toward online learning. Computer access and an internet connection are the general requirements for online learning [34]. However, a proportion of nurses in less-developed areas still does not have access to a computer with an internet connection in the workplace, which hinders their participation in online learning [8,28,35]. This finding suggests that healthcare managers should provide hardware and software support to improve the online learning environment.

In urban hospitals, nurse educators’ attitudes toward online learning were significantly better than clinical nurses and nurse managers. Clinical nurse educators have the responsibility to provide high quality education to nurse students and new nurses, which contributes to producing effective, efficient, and skilled nurses [36]. Therefore, nurse educators have a stronger desire to improve themselves and to keep pace with rapidly updating knowledge, new information technologies, evolving practice requirements, and healthcare expectations. 

With regard to the needs for online learning courses, although there were no significant differences in the needs for each topic, we found that rural nurses reported more learning needs in almost all topics. Furthermore, no matter where nurses work, they ranked nursing communication skills and patient education as the top two topics of interest. Effective communication among healthcare team members is critical for safe and high-quality care [37]. Good interaction between nurses and patients is beneficial in improving health intervention outcomes and patients’ satisfaction [38]. In China, with the continued increase in medical dispute and litigation cases, nurses’ awareness of the importance of communication has also increased [39]. This finding suggests that the design and delivery of continuing learning programs focusing on communication skills should be given priority in the future.

Nurses are important professionals in providing patient education. Nurse-led patient education is beneficial in enhancing patients’ knowledge, understanding, and preparedness for self-management [40]. Although a large amount of education material is available in each hospital, a standardized program of patient education is still lacking. Therefore, an urgent need for patient education methods was reported by nurses. This finding suggests that nursing educators and managers should consider establishing a course to educate patients on theory, method, standardized procedure, and evidence-based content. 

The study also had some limitations. First, although the participants of this study were representative of rural and urban nurses in Shanghai, as one of the most developed cities in China, the result from Shanghai participants may have limitations in generalizing to less-developed regions. Further studies should include samples from less-developed areas in China. Second, the needs for online learning were evaluated by a multiple-choice question. Although the options of question were prepared based on previous studies and local education context, using a validated instrument to confirm our findings in further studies is needed. 

## 5. Conclusions

Health professionals need continuing education to maintain their qualifications and competency. Online learning increases the accessibility and flexibility of continuing education for nurses. The main findings of this study demonstrated that clinical nurses were willing to adopt online learning as a continuing education method. Location of hospital, computer use, and internet access in the workplace, were significantly associated with nurses’ attitudes toward online learning. Rural nurses displayed more positive attitudes towards, and needs for, online learning than those working in urban hospitals. Communication skills and patient education were the most-needed learning topics of nurses. Nursing managers and educators should develop online learning programs and provide appropriate support to fulfill nurses’ learning needs, especially those nurses working in rural healthcare settings. 

## Figures and Tables

**Table 1:** Participants’ demographic and background characteristics.

Variables	*Total (n = 534)*	*Rural (n = 236)*	*Urban (n = 298)*	p *
	*n (%)*	*n (%)*	*n* (%)	
Gender				
Male	5 (0.9)	1 (0.4)	4 (1.3)	0.390
Female	529 (99.1)	235 (99.6)	294 (98.7)	
Age				
≤30 years	333 (62.4)	144 (61.0)	189 (63.4)	0.811
31–39 years	162 (30.3)	75 (31.8)	87 (29.2)	
≥40 years	39 (7.3)	17 (7.2)	22 (7.4)	
Marital status				
Unmarried	253 (47.4)	109 (46.2)	144 (48.3)	0.624
Married	281 (52.6)	127 (53.8)	154 (51.7)	
Educational level				
Diploma or advanced diploma	288 (53.9)	128 (54.2)	160 (53.7)	0.028
Continuing bachelor’s program	187 (35.0)	91 (38.6)	96 (32.2)	
Bachelor of Science and above	59 (11.0)	17 (7.2)	42 (14.1)	
Years of working experience				
≤5 years	211 (39.6)	84 (35.7)	127 (42.6)	0.095
6–9 years	189 (35.4)	95 (40.4)	94 (31.5)	
≥10 years	133 (25.0)	56 (23.8)	77 (25.8)	
*Position*				
First-line nurse	438 (82.0)	198 (83.9)	240 (80.5)	0.604
Nurse educator	43 (8.1)	17 (7.2)	26 (8.7)	
Nurse manager	53 (9.9)	21 (8.9)	32 (10.7)	
Average monthly income (RMB)				
≤5000	186 (34.8)	99 (41.9)	87 (29.2)	0.006
5001–7999	236 (44.2)	89 (37.7)	147 (49.3)	
≥8000	112 (21.0)	48 (20.3)	64 (21.5)	
Computer use and internet access at home				
Yes	525 (98.3)	234 (99.2)	291 (97.7)	0.311
No	9 (1.7)	2 (0.8)	7 (2.3)	
Computer use and internet access in the workplace				
Yes	183 (34.3)	66 (28.0)	117 (39.3)	0.006
No	351 (65.7)	170 (72.0)	181 (60.7)	

* Significance according to chi-squared test between rural and urban hospitals.

**Table 2:** Comparison of attitudes toward online learning between rural and urban nurses.

Attitudes	Total (*n* = 534)	Rural (*n* = 236)	Urban (*n* = 298)	p *
	Mean (*SD*)	Mean (*SD*)	Mean (*SD*)	
Use of computer	14.5 (2.5)	14.7 (2.7)	14.3 (2.4)	0.081
Convenience and flexibility	18.8 (3.0)	19.2 (3.0)	18.5 (2.9)	0.007
Interaction with facilitators and students	26.7 (5.1)	27.5 (5.2)	26.1 (4.9)	0.001
Access to knowledge	8.1 (1.4)	8.2 (1.4)	8.1 (1.5)	0.151
Positive learning experience	25.0 (4.0)	25.8 (4.1)	24.4 (3.7)	<0.001
Improvement of nursing care	11.1 (1.8)	11.3 (1.9)	10.9 (1.8)	0.003
Total score	100.2 (13.6)	102.7 (14.2)	98.3 (12.9)	<0.001

* Significance according to *t*-test between rural and urban hospitals.

**Table 3:** Univariate analysis of association between nurses’ characteristics and attitudes toward online learning.

Variables	*Total (n = 534)*	*Rural (n = 236)*	*Urban (n = 298)*
	*Mean (SD)*	*Mean (SD)*	*Mean (SD)*
Gender			
Male	88.6 (11.3)	81.0 (--)	90.5 (12.1)
Female	100.4 (13.6)	102.8 (14.1)	98.4 (12.9)
*p*	0.055	0.125	0.224
Age			
≤30 years	99.6 (14.0)	103.3 (14.4)	96.8 (13.1)
31–39 years	101.7 (12.3)	102.5 (12.6)	100.9 (12.1)
≥40 years	99.7 (15.4)	98.1 (18.2)	100.9 (13.1)
*p*	0.284	0.352	0.030
Marital status			
Unmarried	99.9 (13.7)	104.1 (14.1)	96.7 (12.5)
Married	100.6 (13.6)	101.5 (14.2)	99.8 (13.1)
*p*	0.537	0.164	0.034
Educational level			
Diploma or advanced diploma	99.4 (53.9)	101.8 (14.3)	97.4 (12.4)
Continuing bachelor’s program	101.2 (14.0)	103.2 (13.5)	99.3 (14.3)
Bachelor of Science and above	101.4 (13.3)	106.0 (16.8)	99.5 (11.3)
*p*	0.300	0.466	0.457
Years of working experience			
≤5 years	100.0 (12.8)	104.1 (14.1)	97.3 (11.2)
6–9 years	98.4 (15.2)	100.8 (15.0)	96.1 (15.1)
≥10 years	103.0 (12.1)	103.6 (12.5)	102.6 (11.9)
*p*	0.012	0.248	0.002
Position			
First-line nurse	99.8 (13.7)	102.9 (14.4)	97.3 (12.4)
Nurse educator	105.2 (9.3)	102.6 (9.1)	106.9 (9.2)
Nurse manager	99.7 (15.7)	100.6 (15.2)	99.0 (16.3)
*p*	0.044	0.782	0.001
Average monthly income (RMB)			
≤5000	100.7 (13.7)	102.8 (15.2)	98.3 (11.6)
5001–7999	100.0 (13.4)	102.8 (13.7)	98.4 (13.1)
≥8000	99.9 (13.9)	102.3 (13.1)	98.2 (14.4)
*p*	0.860	0.978	0.995
Computer use and internet access at home			
Yes	100.45 (13.5)	102.9 (13.9)	98.5 (12.8)
No	88.00 (16.4)	80.0 (28.3)	90.3 (14.1)
*p*	0.006	0.023	0.096
Computer use and internet access in the workplace			
Yes	101.82 (15.1)	106.8 (17.2)	99.0 (13.0)
No	99.42 (12.8)	101.1 (12.5)	97.8 (12.9)
*p*	0.067	0.017	0.409

**Table 4:** Regression analysis on the associated factors of nurses’ attitudes towards online learning.

Variables	Model 1: Overall		Model 2: Rural		Model 3: Urban	
	*B*	*p*	*B*	*p*	*B*	*p*
Gender	NE	NE	NE	NE	NE	NE
Age						
≤30 years (ref.)						
31–39 years	−0.583	0.778	−1.112	0.727	0.885	0.745
≥40 years	−4.361	0.133	−7.198	0.096	−2.956	0.456
Marital *status*						
Unmarried (ref.)						
Married	0.451	0.757	−2.307	0.303	3.077	0.113
Educational level						
Diploma or advanced diploma (ref.)						
Continuing bachelor’s program	2.028	0.155	3.662	0.156	0.593	0.739
Bachelor of Science and above	2.940	0.149	3.567	0.377	2.445	0.288
Years of working experience						
≤5 years (ref.)						
6–9 years	−2.315	0.181	−2.414	0.337	−2.611	0.288
≥10 years	2.203	0.401	2.888	0.464	1.118	0.756
Position						
First-line nurse (ref.)						
Nurse educator	4.943	0.055	0.908	0.823	8.732	0.009
Nurse manager	−0.565	0.787	−3.374	0.337	1.800	0.489
Average monthly income *(RMB)*						
≤5000 (ref.)						
5001–7999	−0.592	0.684	−0.010	0.996	−1.591	0.412
≥8000	−1.760	0.357	−2.030	0.538	−3.490	0.175
Computer use and internet access at home	NE	NE	NE	NE	NE	NE
Computer use and internet access in the workplace						
No (ref.)						
Yes	2.870	0.022	6.542	0.002	0.328	0.832
Location of workplace			NE	NE	NE	NE
Urban (ref.)						
Rural	4.986	<0.001				

*B*: regression coefficient; NE: not entered into the model; ref: reference group.

**Table 5:** Comparison of online learning needs between rural and urban nurses.

Needs	Total (*n* = 534)	Rural (*n* = 236)	Urban (*n* = 298)	p *
	*n* (%)	*n* (%)	*n* (%)	
Specialty nursing	453 (84.8)	208 (88.1)	245 (82.2)	0.058
General nursing	399 (74.7)	182 (77.1)	217 (72.8)	0.256
Nursing skills	440 (82.4)	198 (83.9)	242 (81.2)	0.418
Communication skills	462 (86.5)	211 (89.4)	251 (84.2)	0.082
Patient education	461 (86.3)	211 (89.4)	250 (83.9)	0.065
Nursing informatics	412 (77.2)	182 (77.1)	230 (77.2)	0.986
Nursing management methods	385 (72.1)	171 (72.5)	214 (71.8)	0.869
Nursing education methods	386 (72.3)	169 (71.6)	217 (72.8)	0.757
Nursing research and evidence-based nursing	377 (70.6)	167 (70.8)	210 (70.5)	0.941

* Significance according to chi-squared test between rural and urban hospitals.
